# Dapagliflozin-Associated Euglycemic Diabetic Ketoacidosis in a Patient Who Underwent Surgery for Pancreatic Carcinoma: A Case Report

**DOI:** 10.3389/fsurg.2022.769041

**Published:** 2022-02-23

**Authors:** Xiaoqian Luo, Ran Ji, Weina Lu, Hong Zhu, Libin Li, Jun Hu

**Affiliations:** Department of Surgical Intensive Care Unit, The Second Affiliated Hospital, School of Medicine, Zhejiang University, Hangzhou, China

**Keywords:** DKA, euglycemic diabetic ketoacidosis, dapagliflozin, pancreatic carcinoma, SGLT2i

## Abstract

Diabetic ketoacidosis (DKA), an acute and life-threatening complication of diabetes, is a metabolic disorder caused by insulin deficiency and an increase in counter-regulatory hormones. Several cases of DKA without marked hyperglycemia have been reported and are defined as euglycemic DKA (eu-DKA). The use of sodium-glucose cotransporter 2 inhibitors (SGLT2is) is associated with the occurrence of eu-DKA, of which, dapagliflozin is one of the agents. In this study, we report a case of dapagliflozin-associated eu-DKA following surgery for pancreatic carcinoma. A 57-year-old woman presented with acute abdominal pain after surgery for pancreatic carcinoma. Emergency exploratory laparotomy was performed because of suspicion of gastrointestinal perforation based on a CT scan. The surgeons observed that the stomach was significantly dilated but not perforated. Meanwhile, the patient developed shock and severe acidosis. A further examination confirmed the diagnosis of dapagliflozin-associated eu-DKA. We reviewed the precipitating factors and mechanisms of SGLT2i-associated eu-DKA and discussed the treatment and prevention of this condition. Clinicians need to be alert of the occurrence of SGLT2i-associated eu-DKA in patients treated with this drug in the perioperative period.

## Introduction

Diabetic ketoacidosis (DKA), an acute and life-threatening complication of diabetes, is a metabolic disorder caused by insulin deficiency and an increase in counter-regulatory hormones. As per the United States and United Kingdom guidelines, DKA is usually diagnosed based on hyperglycemia [blood glucose (BG) threshold of 250 or 200 mg/dl], ketosis, and metabolic acidosis (1, 2). However, several cases of DKA have been reported without marked hyperglycemia and are defined as euglycemic DKA (eu-DKA) (1, 3). Because of the absence of hyperglycemia, the diagnosis or exclusion of eu-DKA appears more challenging in emergencies. Generally, the occurrence of eu-DKA is facilitated by the following precipitating factors: strict diet/starvation, pregnancy, alcohol consumption, and use of sodium-glucose cotransporter 2 inhibitors (SGLT2is) (1, 4). Recently, there have been increasing reports of SGLT2i-related Eu-DKA after the use of drugs such as canagliflozin, ipragliflozin, empagliflozin, and dapagliflozin (5–7). However, the use of these agents has not received sufficient attention. Several cases of misdiagnosis or delayed diagnosis have been reported.

Dapagliflozin, an SGLT2i, prevents the reabsorption of glucose from primary urine by inhibiting renal SGLT2 receptors (3, 8). The pathogenesis of euglycemia in SGLT2i-associated eu-DKA is thought to be associated with increased renal glucose clearance by SGLT2is (1, 6). However, eu-DKA does not occur in all patients receiving SGLT2is. More research is required on precipitating factors and mechanisms of SGLT2i-associated eu-DKA.

In this study, we reported a case of dapagliflozin-associated eu-DKA after surgery for pancreatic carcinoma. A 57-year-old woman presented with acute abdominal pain after surgery for pancreatic carcinoma. Emergency exploratory laparotomy was performed because of suspicion of gastrointestinal perforation based on a CT scan. The surgeons observed that the stomach was significantly dilated but not perforated. In addition, the patient developed shock and severe acidosis. Further examination confirmed the diagnosis of dapagliflozin-associated eu-DKA. We report this case to remind surgeons to be alert of the occurrence of SGLT2i-associated eu-DKA in patients receiving this medication during the perioperative period.

## Case Report

**Figure 1:**
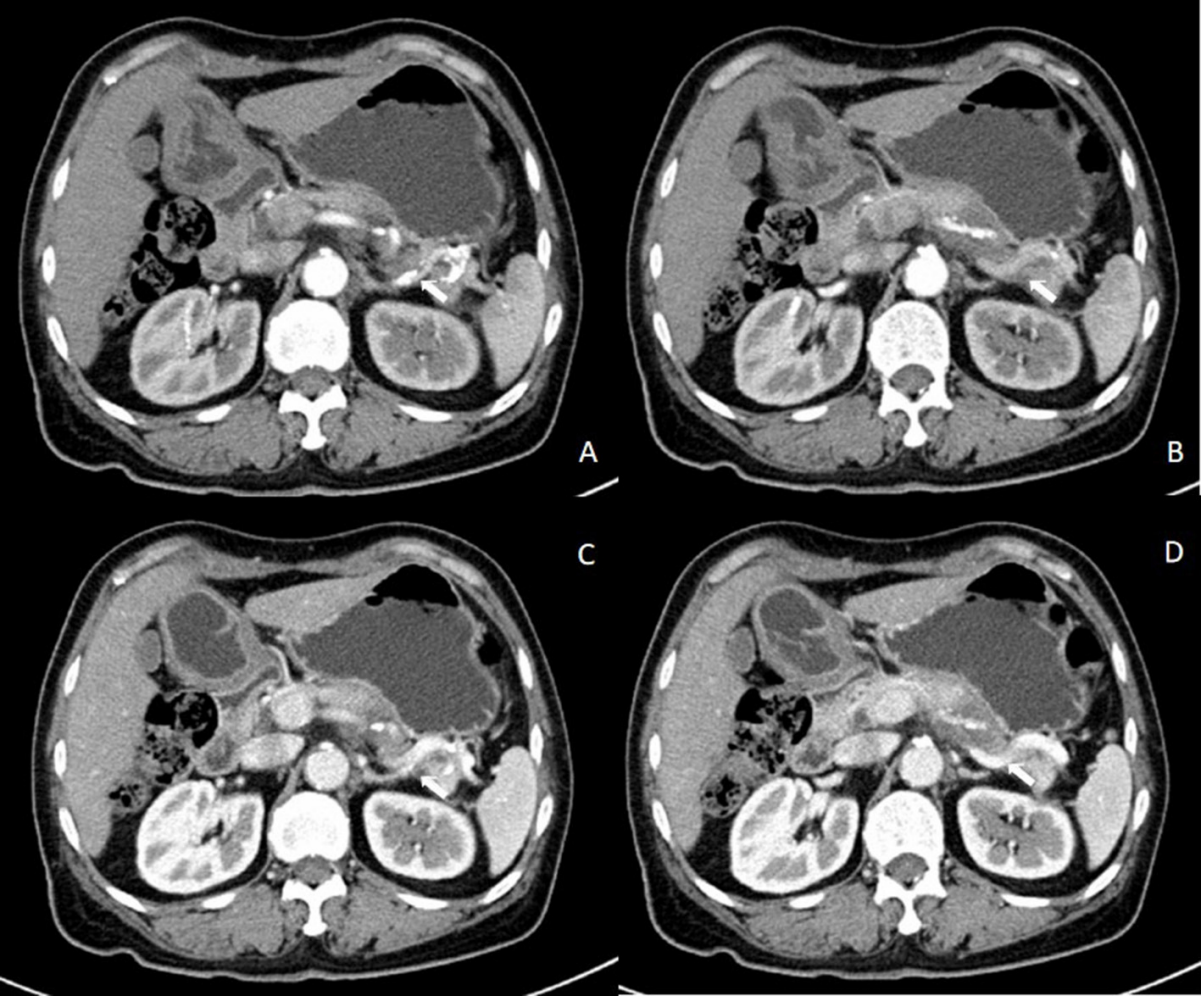
Results of contrast-enhanced abdominal CT before the first surgery. Contrast-enhanced abdominal CT in the **(A,B)** arterial phase and in the **(C,D)** venous phase showing an enhanced mass on the tail of the pancreas invading the splenic vessel.

**Figure 2:**
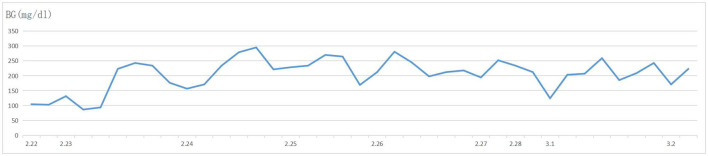
Blood glucose levels of the patient after resection of the pancreatic carcinoma. The level of blood glucose consistently remained between 86 and 295 mg/dl after resection of the pancreatic carcinoma.

**Figure 3:**
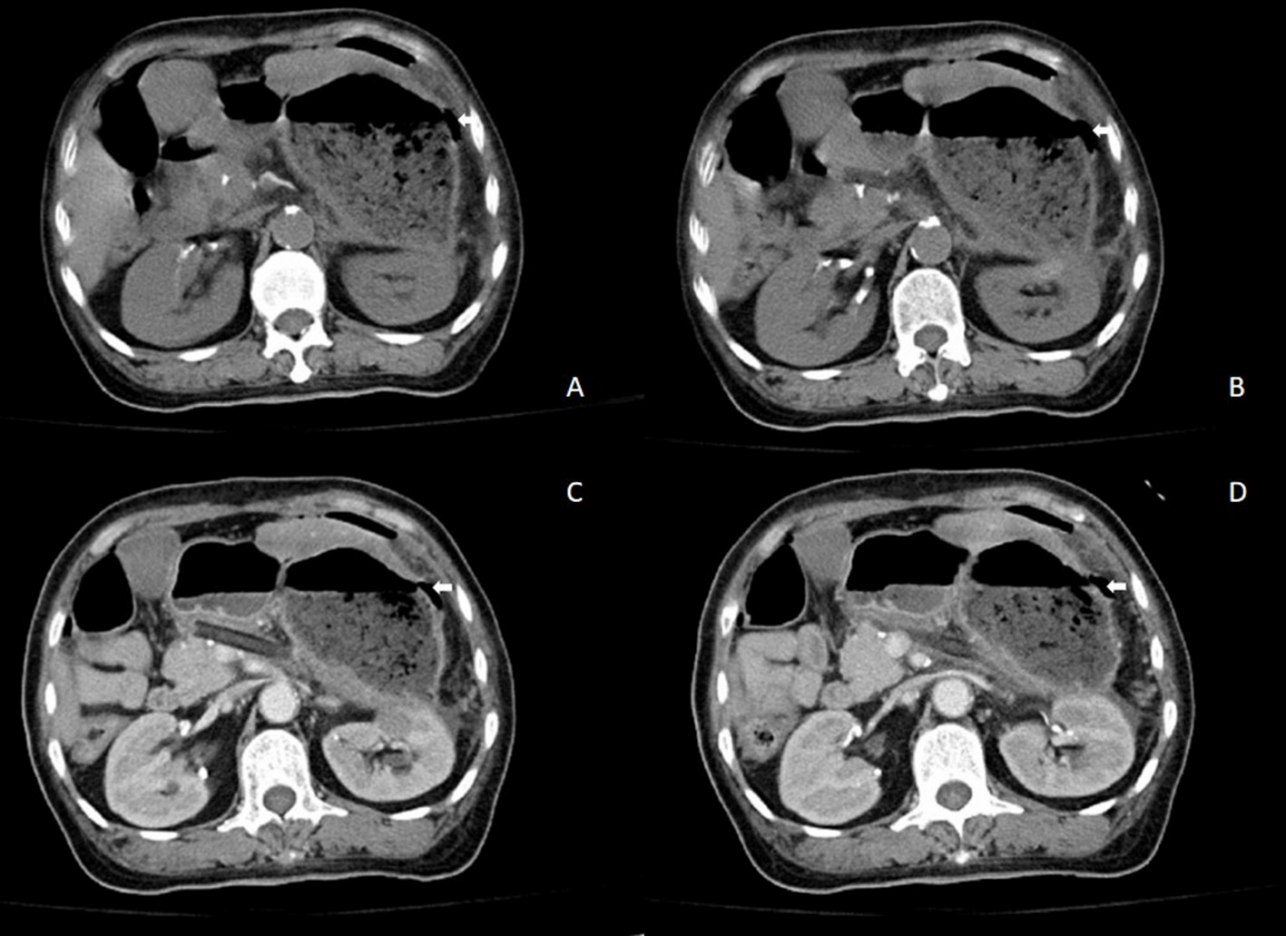
Results of contrast-enhanced abdominal CT when the patient suddenly presented with acute abdominal pain. **(A,B)** Abdominal CT and **(C,D)** contrast-enhanced abdominal CT showing free intraperitoneal air and suspected discontinuity of the wall of greater gastric curvature.

A 57-year-old woman was admitted to a hospital with upper abdominal pain for more than half a year. The pain was mild. Physical examination of the patient did not reveal any pathological findings. The patient had been taking appropriate oral medications for diabetes mellitus, hypertension, and stroke for the past 6 years. The oral agents prescribed to the patient at the time of presentation were metformin (0.5 g twice daily), dapagliflozin (5 mg once daily), and aspirin (100 mg once daily). Contrast-enhanced CT revealed an enhanced mass on the tail of the pancreas invading the splenic vessel, which was diagnosed as pancreatic carcinoma (Figure 1). The clinical stage was T2N0M0 according to the American Joint Committee on Cancer tumor node metastasis (TNM) staging (8th edition). Laparoscopic distal pancreatectomy with *en bloc* splenectomy was conducted. Short gastric arteries were ligated. A hard grayish-white mass of about 4 cm in diameter was observed on the tail of the pancreas, and pathological investigations revealed a poorly differentiated pancreatic ductal adenocarcinoma. Somatostatin was routinely used to suppress pancreatic secretion after surgery. The patient started a clear liquid diet on the third postoperative day. Meanwhile, metformin (0.5 g twice daily) and dapagliflozin (5 mg once daily) were reinitiated, and blood glucose level was monitored. The patient only experienced slight back and incision site pain. Daily drainage volume consistently remained between 10 and 80 ml. The amylase level of the drainage fluid was 50,600 U/L on the 3rd postoperative day. The postoperative pancreatic fistula was diagnosed according to the definition of the International Study Group of Pancreatic Surgery. However, the patient had a sudden onset of severe postprandial abdominal pain on the 8th postoperative day. Physical examination showed abdominal distention, mild tenderness, and suspicious rebound tenderness. The patient had no chills or fever, and her vital signs were stable. A small amount of bloody fluid was drained from the abdominal drainage tube. Peripheral blood investigation revealed a white blood cell (WBC) count of 16,000 μl (neutrophils 87.8%). Serum biochemical examination revealed the following: glucose 223 mg/dl, total bilirubin 8.6 umol/L, alanine aminotransferase (ALT) 9 U/L, albumin 3.61 g/dl, and C-reactive protein 168.1 mg/L. Postoperative BG levels of the patient consistently remained between 86 and 295 mg/dl (Figure 2). Emergency contrast-enhanced CT revealed free intraperitoneal air and suspected discontinuity of the wall of greater gastric curvature (Figure 3). Considering the possibility of gastric perforation, an emergency exploratory laparotomy was performed. However, the surgeons observed that the stomach was significantly dilated but not perforated. Blood supply to the stomach wall was normal, and there was no necrosis in any part of the stomach. Meanwhile, the patient developed shock with severe acidosis (pH 6.8). The patient was transferred to the surgical intensive care unit after the emergency surgery. Arterial blood gas analysis revealed pH of 7.09, partial pressure of carbon dioxide of 56.7 mmHg, ${\text{HCO}}_{3}^{-}$ of 14 mmol/L, and anion gap of 36.2 mmol/L, indicating high anion gap metabolic acidosis with respiratory acidosis. The patient's temperature was 36.5°C, blood pressure was 114/61 mmHg (with norepinephrine, 0.13 ug/kg^*^min), and heart rate was 125 bpm. Peripheral blood investigation revealed a white blood cells (WBC) count of 26,800 μl (neutrophils 87.5%). Serum biochemical examination revealed the following: glucose, 270 mg/dl, procalcitonin 2.66 ng/ml, total bilirubin 8.9 umol/l, ALT 28U/l, albumin 3.33 g/dl, and C-reactive protein 137.8 mg/L. Based on the results of serum biochemical investigations, symptoms, medical history, and surgical findings, we suspected eu-DKA, which misguided the surgeons into performing exploratory laparotomy. Then, blood beta-hydroxybutyrate (BOHB) was revealed to be 10.87 mmol/L (normal range 0.03-0.3 mmol/L), and urine ketones were strongly positive (4+). These results confirmed our diagnosis. Through literature review, we identified dapagliflozin as the possible cause of eu-DKA. Dapagliflozin was discontinued, and the patient underwent fluid replacement and insulin therapy. One day later, the shock and severe acidosis were corrected, and the endotracheal tube was removed. Four days later, the patient was transferred to a regular room. The patient was treated with nutritional support, suppression of pancreatic secretion, and insulin to control blood glucose. The patient recovered and was discharged on the 42nd day of hospitalization.

## Discussion

Hyperglycemia, ketosis, and metabolic acidosis are classic clinical manifestations of DKA. In 1973, Munro et al. reported 211 episodes of DKA in patients with type 1 diabetes. They found that 37 patients had a BG level of <300 mg/L, and that 16 patients had a level of <200 mg/L. They described this condition as “euglycemic DKA” for the first time (9). Subsequently, in 1993, Jenkins et al. reported a case series of 722 episodes of DKA in Birmingham, United Kingdom. A total of 23 patients were diagnosed with eu-DKA, and eight patients had a BG level of <200 mg/L (10). The DKA, without marked hyperglycemia, is the main feature of eu-DKA. Traditional causes of DKA without marked hyperglycemia vary and include strict diet, starvation, pregnancy, alcohol consumption, and chronic liver disease (1, 4, 11, 12). The SGLT2is was first marketed in 2013 as a newest class of antihyperglycemic medication. In 2015, several studies reported that SGLT2is might predispose ketoacidosis (13). A few cases treated with SGLT2is demonstrated an eu-DKA (14–16). However, SGLT2i-associated eu-DKA has not attracted much attention.

More research is required on precipitating factors and mechanisms of SGLT2i-associated eu-DKA. Insulin deficiency and increase in glucagon levels are usually considered mechanisms of SGLT2i-associated DKA (1). SGLT2 is expressed by human pancreatic alpha cells. Several studies have reported that SGLT2is might have a direct effect on pancreatic alpha cells (1, 17). Recently, Capozzi et al. demonstrated that SGLT2i-associated DKA was not altered by interruption of glucagon signaling and stated that the condition might occur independently of glucagon (18).

Approximately one-third of patients with SGLT2i-associated DKA demonstrate lower BG levels or “euglycemic” status (19). An increase in renal glucose clearance may have contributed to the lower BG levels of patients with SGLT2i-associated DKA (1). Starvation, surgery, acute illness, dehydration, and excessive alcohol intake may be precipitating factors of unremarkable hyperglycemia in SGLT2i-associated DKA. Several studies have reported the occurrence of SGLT2i-associated eu-DKA in patients with acute pancreatitis (15, 20). In our case report, the surgery and the perioperative management might be related to the occurrence of SGLT2-iassociated eu-DKA. However, the question remains whether eu-DKA was precipitated by the insufficiently drained pancreatic fistula, secondary inflammation after surgery, or the pancreatic malignancy itself. Further research is required.

Clinical features of eu-DKA are similar to those of DKA. Classic clinical presentation includes dehydration, tachypnea, deep and sighing (Kussmaul) respiration, nausea, vomiting, abdominal pain that could mimic an acute abdominal condition, confusion, drowsiness, and loss of consciousness (1, 21). Biochemical criteria for the diagnosis of DKA are hyperglycemia (BG threshold of 250 or 200 mg/dl), metabolic acidosis (venous pH <7.3 or serum bicarbonate <15 mmol/L), and ketonemia or ketonuria. Blood BOHB concentration is usually ≥ 3 mmol/L. Urine ketones are typically moderate or largely positive (≥ 2+) (21). The level of BG in eu-DKA is usually normal or unremarkable (<200 or 250 mg/dl). Unremarkable BG level may be the only difference between eu-DKA and DKA, which also makes it challenging to diagnose the disease.

Conventional treatment of SGLT2i-associated eu-DKA includes rehydration, insulin therapy, and glucose administration, and is similar to that of DKA. The mainstay of treatment is rehydration, which can normalize the volume and increase the clearance of ketone bodies (22). Insulin can decrease the synthesis of liver glucose and ketone bodies and increase the utilization of ketone bodies. Moreover, low insulin concentrations can affect lipolysis (22). Therefore, insulin administration should be continued even when the level of BG decreases below 200 mg/dl. Preventing the occurrence of SGLT2i-associated eu-DKA appears to be more important than its treatment. The SGLT2is should be avoided or withheld in the presence of the precipitating factors of SGLT2i-associated eu-DKA. It is recommended to use insulin instead of SGLT2is to control BG. The BG level and arterial blood gas of patients with pancreatic disease should be monitored, especially during the perioperative period.

## Conclusion

Euglycemic diabetic ketoacidosis (eu-DKA) is a life-threatening disease, and the diagnosis of and ability to distinguish the condition are challenging during an emergency because of unremarkable hyperglycemia. A case of dapagliflozin-associated eu-DKA has been reported in the present study, which was associated with abdominal pain that mimicked acute surgical abdominal pain after surgery for pancreatic carcinoma. Awareness should be raised when patients are receiving this medication in the perioperative period, because eu-DKA is a potentially life-threatening complication. Further research is required on precipitating factors and mechanisms of SGLT2i-associated eu-DKA. Moreover, prevention of the occurrence of SGLT2i-associated eu-DKA appears to be more important than treatment.

## Data Availability Statement

The original contributions presented in the study are included in the article/supplementary material, further inquiries can be directed to the corresponding author.

## Ethics Statement

The study were reviewed and approved by appropriate institutional of the Second Affiliated Hospital, School of Medicine, Zhejiang University. The patients/participants provided their written informed consent to participate in this study. Written informed consent was obtained from the individual(s) for the publication of any potentially identifiable images or data included in this article.

## Author Contributions

JH, XL, and RJ collected the data and drafted the manuscript. WL, HZ, and LL reviewed and modified the manuscript. All authors agreed on the final version.

## Conflict of Interest

The authors declare that the research was conducted in the absence of any commercial or financial relationships that could be construed as a potential conflict of interest.

## Publisher's Note

All claims expressed in this article are solely those of the authors and do not necessarily represent those of their affiliated organizations, or those of the publisher, the editors and the reviewers. Any product that may be evaluated in this article, or claim that may be made by its manufacturer, is not guaranteed or endorsed by the publisher.

## Abbreviations

DKA: diabetic ketoacidosis
eu-DKA: euglycemic diabetic ketoacidosis
SGLT2i: sodium-glucose co-transporter 2 inhibitors
BG: blood glucose
ALT: alanine aminotransferase
NE: norepinephrine
WBC: white blood cell.
